# Association Analysis Provides Insights into Plant Mitonuclear Interactions

**DOI:** 10.1093/molbev/msae028

**Published:** 2024-02-07

**Authors:** Qun Lian, Shuai Li, Shenglong Kan, Xuezhu Liao, Sanwen Huang, Daniel B Sloan, Zhiqiang Wu

**Affiliations:** Shenzhen Branch, Guangdong Laboratory for Lingnan Modern Agriculture, Genome Analysis Laboratory of the Ministry of Agriculture, Agricultural Genomics Institute at Shenzhen, Chinese Academy of Agricultural Sciences, Shenzhen 518120, China; Shenzhen Branch, Guangdong Laboratory for Lingnan Modern Agriculture, Genome Analysis Laboratory of the Ministry of Agriculture, Agricultural Genomics Institute at Shenzhen, Chinese Academy of Agricultural Sciences, Shenzhen 518120, China; Key Laboratory of Biology and Genetic Improvement of Horticultural Crops of the Ministry of Agriculture, Sino-Dutch Joint Laboratory of Horticultural Genomics, Institute of Vegetables and Flowers, Chinese Academy of Agricultural Sciences, Beijing 100081, China; Shenzhen Branch, Guangdong Laboratory for Lingnan Modern Agriculture, Genome Analysis Laboratory of the Ministry of Agriculture, Agricultural Genomics Institute at Shenzhen, Chinese Academy of Agricultural Sciences, Shenzhen 518120, China; Marine College, Shandong University, Weihai 264209, China; Shenzhen Branch, Guangdong Laboratory for Lingnan Modern Agriculture, Genome Analysis Laboratory of the Ministry of Agriculture, Agricultural Genomics Institute at Shenzhen, Chinese Academy of Agricultural Sciences, Shenzhen 518120, China; Shenzhen Branch, Guangdong Laboratory for Lingnan Modern Agriculture, Genome Analysis Laboratory of the Ministry of Agriculture, Agricultural Genomics Institute at Shenzhen, Chinese Academy of Agricultural Sciences, Shenzhen 518120, China; State Key Laboratory of Tropical Crop Breeding, Chinese Academy of Tropical Agricultural Sciences, Haikou 571101, China; Department of Biology, Colorado State University, Fort Collins, CO 80523, USA; Shenzhen Branch, Guangdong Laboratory for Lingnan Modern Agriculture, Genome Analysis Laboratory of the Ministry of Agriculture, Agricultural Genomics Institute at Shenzhen, Chinese Academy of Agricultural Sciences, Shenzhen 518120, China; Shenzhen Branch, Guangdong Laboratory of Lingnan Modern Agriculture, Key Laboratory of Synthetic Biology, Ministry of Agriculture and Rural Affairs, Agricultural Genomics Institute at Shenzhen, Chinese Academy of Agricultural Sciences, Shenzhen 518120, China

**Keywords:** mitonuclear coevolution, mitochondria, linkage disequilibrium, RNA editing, association analysis

## Abstract

Cytonuclear interaction refers to the complex and ongoing process of coevolution between nuclear and organelle genomes, which are responsible for cellular respiration, photosynthesis, lipid metabolism, etc. and play a significant role in adaptation and speciation. There have been a large number of studies to detect signatures of cytonuclear interactions. However, identification of the specific nuclear and organelle genetic polymorphisms that are involved in these interactions within a species remains relatively rare. The recent surge in whole genome sequencing has provided us an opportunity to explore cytonuclear interaction from a population perspective. In this study, we analyzed a total of 3,439 genomes from 7 species to identify signals of cytonuclear interactions by association (linkage disequilibrium) analysis of variants in both the mitochondrial and nuclear genomes across flowering plants. We also investigated examples of nuclear loci identified based on these association signals using subcellular localization assays, gene editing, and transcriptome sequencing. Our study provides a novel perspective on the investigation of cytonuclear coevolution, thereby enriching our understanding of plant fitness and offspring sterility.

## Introduction

Since the endosymbiotic origin of mitochondria and plastids, they have transitioned from independent bacteria into semiautonomous organelles through a complex and ongoing process of cytonuclear integration (Adams et al. 2002; Timmis et al. 2004; Kleine et al. 2009; Keeling 2010; Johnston and Williams 2016; Roger et al. 2017; Sloan et al. 2018; Wang et al. 2024). Many important cell functions occur in these organelles, including cellular respiration, photosynthesis, lipid metabolism, and synthesis of nucleotides and amino acids (Millar et al. 2005; Wijk and Baginsky 2011; Forsythe et al. 2019). However, organelle genomes contain only a small proportion of the genes required for these biochemical functions. For example, the plastid genome typically contains ∼100 to 120 genes, and the mitochondrial genome only contains 19 to 60 genes in land plants (Tonti-Filippini et al. 2017; Mower 2020; Kan et al. 2021). Hence, most organelle proteins are encoded by the nuclear genome and transported into organelles (Millar et al. 2005; Wijk and Baginsky 2011; Lee and Millar 2016). Even though nuclear and organelle genomes are retained in the same cell, they differ in many key properties such as mode of inheritance, evolutionary rate, and genome copy number (Mogensen 1996; Lynch et al. 2006; Smith and Keeling 2015). Therefore, coevolution between nuclear and organelle genomes has been a longstanding area of interest.

Cytonuclear coevolution can play a significant role in reproductive isolation and adaptive evolution. Cytonuclear incompatibilities can lead to reduced fitness or even sterility in hybrid offspring (Chase 2007; Burton and Barreto 2012; Barnard-Kubow et al. 2016; Chen et al. 2017; Sloan et al. 2017; Fishman and Sweigart 2018). For example, when the wild pea (*Pisum sativum* subsp. *elatius*) was used as the female parent in crosses with its domesticated relative, almost all F1 hybrids were sterile and showed chlorophyll deficiency. In contrast, when the wild pea was used as the male parent, the hybrids had a normal phenotype (Bogdanova et al. 2009). Such asymmetries in reciprocal crosses often reflect effects of cytoplasmic genetics and cytonuclear interactions (Turelli and Moyle 2007; Burton and Barreto 2012; Sloan et al. 2017). Furthermore, crossing experiments have shown that different combinations of nuclear restorer-of-fertility genes and mitochondrial chimeric open reading frames (ORFs) are the genetic basis of cytoplasmic male sterility (CMS), which is used in hybrid breeding for numerous important crop species (Kim and Zhang 2018; Takatsuka et al. 2021; Wanga et al. 2021; Jiang et al. 2022). Cytoplasmic genetics and cytonuclear interactions may also be significant for hybrid offspring adapting to new habitats. For example, hybridization between *Helianthus annuus* (xeric habitat) and *Helianthus petiolaris* (mesic habitat) and subsequent experimental transplants showed that parental cytoplasms were locally adapted and that hybrid success was affected by cytonuclear interactions (Lexer et al. 2003; Sambatti et al. 2008). Therefore, identifying signatures of cytonuclear coevolution involving nuclear genes that functionally interact with organelle gene products is an important goal.

There are already many methods and extensive datasets for identification of nuclear-encoded proteins that are targeted to the mitochondria and plastids (Emanuelsson et al. 2000; Hooper et al. 2017; Sperschneider et al. 2017; Forsythe et al. 2019). However, identifying genes that are involved in cytonuclear interaction and coevolution is more challenging. In some cases, mapping of genes responsible for hybrid incompatibilities has identified specific loci involved in cytonuclear epistasis (Sackton et al. 2003; Lee et al. 2008; Meiklejohn et al. 2013; Moran et al. 2024), but the number of successful examples of this approach remains limited. Phylogenetic analysis of evolutionary rate covariation (ERC) has been another way to identify proteins that are functionally related and/or coevolving (Findlay et al. 2014; Raza et al. 2019; Yan et al. 2019; Forsythe et al. 2021). ERC is based on the principle that functional interactions between proteins should lead to correlated changes in evolutionary rates across a phylogeny that samples diverse species. Genetic diversity within species or in hybrid populations has also been analyzed to scan for coadapted combinations of nuclear and cytoplasmic variants, based on maintenance of associations (linkage disequilibrium [LD]) between compatible alleles (Bar-Yaacov et al. 2015; Sloan et al. 2015; Evans et al. 2021; Ward et al. 2022; Moran et al. 2024). However, because nuclear and cytoplasmic loci are unlinked, such associations are expected to be difficult to detect except in the presence of strong selection against cytonuclear incompatibilities (Eyre-Walker 2017).

Genome-wide association studies (GWAS) are typically used to find association relationships between nuclear genetic polymorphisms and phenotypic variation within a species, including traits related to mitochondrial function. For example, Hodgkinson et al. (2014) found that variation in posttranscriptional modifications of human mitochondrial tRNAs could be explained by genetic variation at specific nuclear loci. In addition, variation in mitochondrial DNA copy number and heteroplasmy levels in humans was recently associated with nuclear variants (Gupta et al. 2023). Conceptually, similar approaches can be used in genome-wide searches for nuclear loci associated with mitochondrial DNA polymorphisms. For example, a correlation between mitochondrial DNA haplotypes and a nuclear genome region that contains genes with mitochondrial functions was found in hybridizing warblers (Wang et al. 2021). In addition, Wang, Li, et al. (2022) identified nuclear restorer-of-fertility genes associated the mitochondrial chimeric ORFs in *Citrus* by using a similar approach. The growing availability of population genomic data from many species gives us an opportunity to test for the effects of selection in maintaining mitonuclear LD.

In this study, we identified mitochondrial genome variants in flowering plants and detected mitonuclear LD with a GWAS-like approach. We then investigated the signatures of the association genomic regions with the potential mitochondrial function. Moreover, we investigated examples of nuclear loci identified by our method through subcellular localization assays, gene editing, and transcriptome sequencing.

## Results

### Detecting Variants in the Mitochondrial Genome

To explore the association between mitochondrial and nuclear genomes (hereafter referred as mitonuclear LD), we selected 7 species that exhibit a wide range of mitochondrial genome size (from 365 Kb to 2.9 Mb) and include both eudicots and monocots (supplementary table S1, Supplementary Material online). To identify sequence variants across the mitochondrial genome in each species, we obtained published whole genome sequencing (WGS) data from NCBI, including the sequences of plastid, mitochondrial, and nuclear genomes. These sampled species were *Arabidopsis thaliana* (arabidopsis), *Oryza sativa* (rice), *Zea mays* (maize), *Solanum lycopersicum* (tomato), *Cucumis melo* (melon), *Cucumis sativus* (cucumber), and *Citrullus lanatus* (watermelon) (Qi et al. 2013; Lin et al. 2014; Alonso-Blanco et al. 2016; Bukowski et al. 2018; Wang et al. 2018; Guo et al. 2019; Zhao et al. 2019) (supplementary table S2, Supplementary Material online). Reads from between 115 and 1,184 (supplementary table S2, Supplementary Material online) individuals from each species were mapped against the corresponding mitochondrial reference genome to detect polymorphic sequence variants.

To ensure the reliability of identified mitochondrial single-nucleotide polymorphisms (SNPs) and indels, we applied strict filters to avoid false positives caused by duplicate regions within the mitochondrial genome or regions shared between the mitochondrial genome and nuclear or plastid genomes due to intracellular gene transfer. Sequencing depth of the mitochondrial genome ranged from 98.88 to 196.86 with an average of 127.61 across the 7 species (supplementary table S1, Supplementary Material online). After rigorous variant calling and filtering (see the Materials and Methods section for details), we obtained a final set of 624 to 17,578 (mean = 4,148) mitochondrial variants per species (supplementary table S1, Supplementary Material online). Overall, 82.3% of mitochondrial variants consisted of SNPs, with the majority of indels being <5 bp in size (supplementary fig. S1, Supplementary Material online). On average, 20.85% of the mitochondrial variants were located in coding regions, with melon being an outlier (4.89%) possibly due to its large mitochondrial genome (2.9 Mb), which has an unusually low proportion of genic content (supplementary table S1, Supplementary Material online). The nucleotide diversity of mt-genome (mitochondrial genome) also varies in different species (supplementary fig. S2, Supplementary Material online).

### Nuclear Loci Exhibiting Mitonuclear LD by GWAS Analysis

**Fig. 1.**
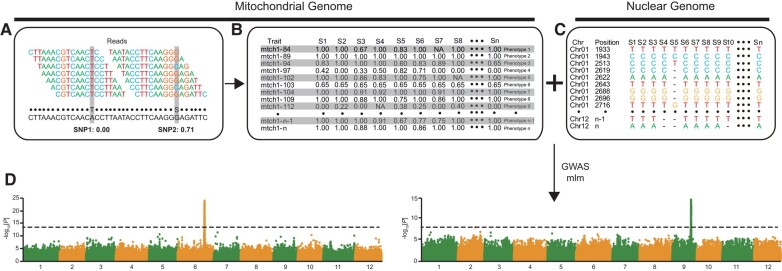
Use of mitochondrial variants as “phenotypes” in GWAS analysis. a) The identification of the reference allele frequency involves calculating the read ratio that supports the reference genotype for mitochondrial variations subjected to strict filtering criteria. For instance, in the case of SNP1, the reference allele ratio is 0/7, and for SNP2, it is 5/7. A black dot indicates a genotype matching the reference genome. Heterozygous loci are annotated with degenerate bases. b) Convert the reference allele frequency of mitochondrial variations into a phenotype matrix, treating each mitochondrial variation as an individual phenotype. c) The nuclear genome variants involved in GWAS analysis and the diagram of the result (d) using mlm model.

GWAS have proven to be a powerful tool for investigating the association relationship between genotype and phenotype. Here, we adapted the approach of Hodgkinson et al. (2014) (Fig. 1) to explore mitonuclear LD and the potential for coevolution between the mitochondrial and nuclear genomes (Burton et al. 2013).

We detected significant mitonuclear association signals in all the species studied, but the number of associations differed substantially (Table 1). The highest number of associations was found in melon with 290, followed by cucumber with 283 associations. Even though it possesses fewer mitochondrial variants, maize exhibited a similar number of associations (228) compared with melon and cucumber. Conversely, tomato had many mitochondrial variants (supplementary table S1, Supplementary Material online) but a lower number of associations (40), even compared with rice (88) and watermelon (69). The lowest number of associations was found in arabidopsis with only 15. The total number of nuclear genes found in regions exhibiting associations with at least 1 mitochondrial variant also varied from species to species, ranging from 1,838 in cucumber to 71 in arabidopsis (supplementary table S3, Supplementary Material online). We also observed the phenomenon that closely linked mitochondrial variants can be linked to the same nuclear region (supplementary fig. S3, Supplementary Material online).

**Table 1:** The summary of associated signals and genes

Species	Associated signals^a^	Associated genes^b^	Percentage^c^
Arabidopsis	15	71	0.26%
Rice	88	955	2.51%
Maize	228	1,662	3.78%
Tomato	40	284	0.79%
Watermelon	69	479	2.12%
Cucumber	283	1,838	7.57%
Melon	290	1,601	5.84%

^a^Number of signals identified in association analysis. ^b^Genes encompassed by GWAS peaks. ^c^Percentage of associated genes out of all the annotated genes within each species.

As an example to illustrate the identified patterns of mitonuclear LD, we detected a significant signal on rice chromosome 12 associated with a mitochondrial genome SNP (AB076665:248850) (supplementary fig. S4a, Supplementary Material online). The most significant SNP in this nuclear region was located in a gene (*OsNDPK2*) encoding a protein that was predicted to be targeted to both mitochondria and plastids and involved in abiotic stress response and plastid development (Ye et al. 2016). As another example, we observed an association signal related to an arabidopsis mitochondrial SNP (NC_037304:120773) with the most significant nuclear SNP located in a gene (*AT1G10760*) known to play an important role in environmental adaptation such as cold acclimation and response to symbiotic fungus, as well as in starch catabolic process (supplementary fig. S4b, Supplementary Material online; Yu et al. 2001). The protein expressed by *AT1G10760* is predicted to be targeted to both mitochondria and plastids (https://www.arabidopsis.org/).

CMS systems serve as an outstanding model for investigating mitonuclear interaction in plants (Toriyama 2021). In our study, we also identified a pentatricopeptide repeat (PPR) gene annotated as a fertility restorer (*Rf3*) (Xu et al. 2009) in maize (supplementary fig. S5, Supplementary Material online). In summary, the successful identification of these genes associated with mitonuclear interactions provides some validation for the potential of this GWAS approach.

### Characterization of Genes Exhibiting Mitonuclear LD

We hypothesized that a history of selection on mitonuclear interactions may have been responsible for signals of mitonuclear LD. However, such selection may indirectly affect multiple genes in a region due to LD blocks in the nuclear genome (Korte and Farlow 2013; Liu and Yan 2019). Despite the presence of other nuclear genes with potentially unrelated functions, we predicted that regions exhibiting mitonuclear LD would be statistically enriched for nuclear genes with known mitochondrial functions. To test this prediction, we performed a global Gene Ontology (GO) enrichment analysis. We observed mitochondrial-related GO terms in 6 of the studied species. The only exception was arabidopsis, which might have been due to the small sample of regions identified with mitonuclear LD in this species. These GO terms include “mitochondrial transmembrane transport”, “protein targeting to mitochondrion” and “mitochondrial mRNA processing” (supplementary table S4, Supplementary Material online). Generally, the number of GO terms related to mitochondria increased with the number of regions exhibiting mitonuclear LD. For example, we detected the highest number of enriched mitochondrial-related GO terms in maize and melon, both of which had a large number of regions with mitonuclear LD. However, this pattern did not always hold true, as we identified only 1 GO term related to mitochondrial function in cucumber, even though it had a high number of regions detected in mitonuclear LD analysis (supplementary table S4, Supplementary Material online).

**Fig. 2.**
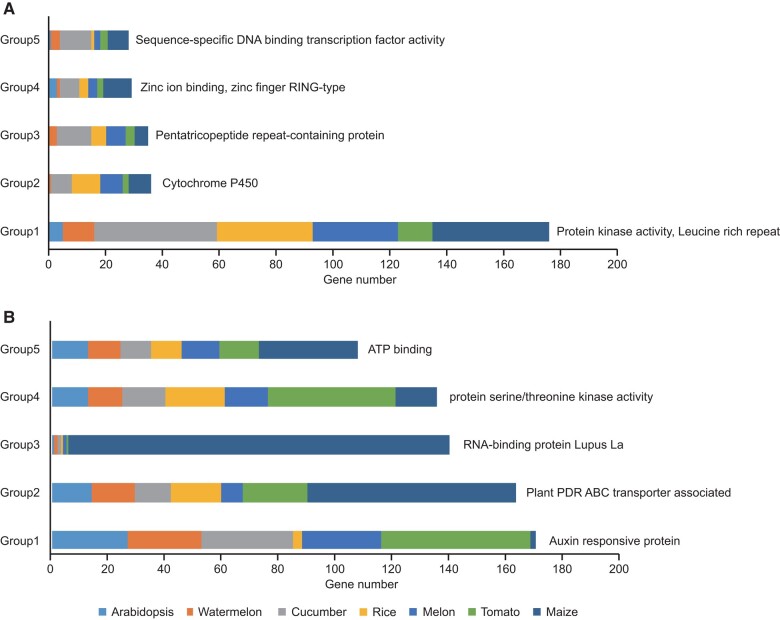
Functional classification of the top 5 orthogroups from genes found in GWAS peaks. Stacking histogram of orthogroups generated from the subset of genes identified in GWAS peaks (a) and all genes (b).

To identify genes and gene families that exhibited mitonuclear LD in multiple species, we clustered all 6,890 nuclear genes found in our analysis into 1,080 orthogroups using OrthoFinder2 (Emms and Kelly 2019). Sixty-two of these orthogroups contained at least of 5 of the 7 species in our analysis. The third largest orthogroup consisted of PPR proteins (Fig. 2a), which are known to have diverse roles in mitochondria, such as controlling intron splicing and RNA editing (Lurin et al. 2004; Xiao et al. 2018; Ren et al. 2020). PPR proteins are also important in CMS breeding systems because of their role in suppressing or counteracting CMS ORFs (Wise and Pring 2002).

Some of the other largest orthogroups also contained genes with mitochondrial-related functions. For example, cytochrome P450 enzymes (the second largest orthogroups) are targeted to mitochondria and can alter catalytic activities using electrons from the mitochondrial electron transport system (Ahn and Yun 2010). An extensive relationship between mitochondria and the leucine-rich repeat kinase gene family (the largest orthogroups) is not well established in plants, but the leucine-rich repeat kinase 2 gene (*LRRK2*) has been found to associate with the mitochondrial outer membrane and affect Parkinson’s disease in humans (West et al. 2005). As a comparison, to assess whether these functional categories were enriched solely because they are most abundant in these genomes, we repeated this analysis using all the genes from each of the 7 species. In this control analysis, none of the aforementioned functional categories appeared in the 5 largest orthogroups (Fig. 2b).

**Fig. 3.**
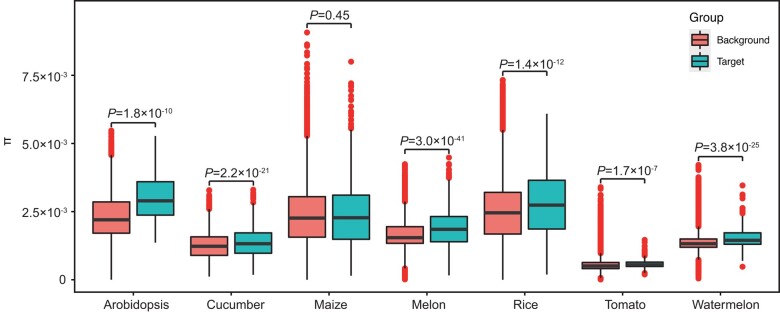
Contrasting levels of nucleotide polymorphism between target and background genes in 7 species. “Target” indicates nuclear genes exhibiting mitonuclear association, while “background” indicates the other genes in the nuclear genome. Central bold lines indicate the median, and box limits represent the upper and lower quartiles. Whiskers extend to data no more than 1.5 times the interquartile range, and dots represent outlier data points. Significant differences were determined by 2-sided Wilcoxon rank sum test.

To test whether nuclear genes found in peaks of mitonuclear LD displayed different patterns of selection or accelerated evolution relative to the rest of the genome, we calculated nonsynonymous/synonymous substitution ratios (Ka/Ks). This analysis did not detect any significant differences in Ka/Ks (2-sided Wilcoxon rank sum test) for any of the species in this study. However, we did find that associated genes exhibited higher nucleotide polymorphism (π) than the rest of the nuclear genes in all species except for maize (Fig. 3).

### Candidate Genes That May Influence Mitonuclear LD

**Fig. 4.**
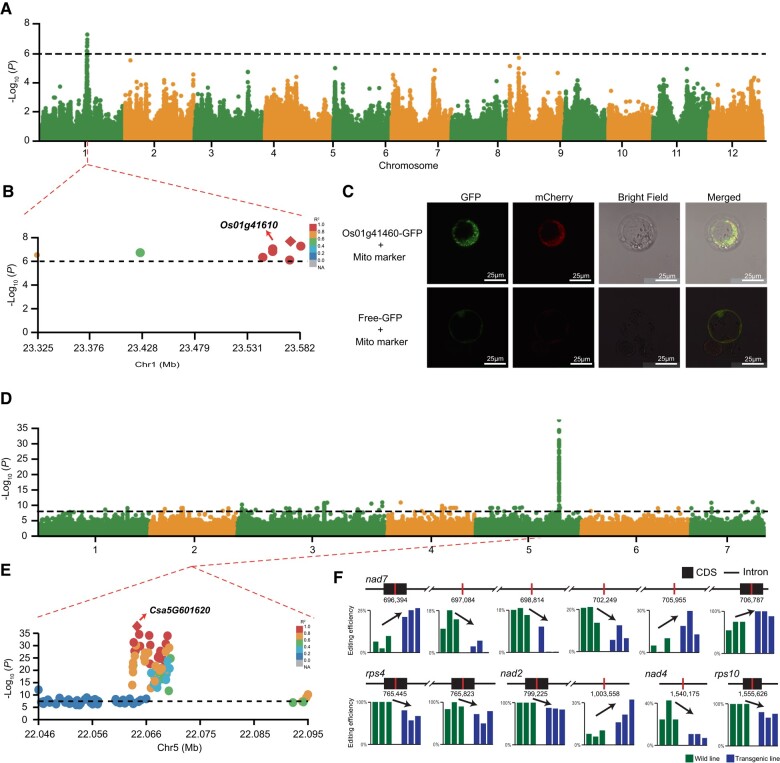
Identification of candidate genes involved in mitonuclear interaction. a, b) Manhattan plot of GWAS analysis of mitochondrial SNP (AB076665: 324769) in rice. The dashed horizontal line indicates the significance threshold. Only the SNPs exceeding this threshold were drawn in the zoomed-in plot, and the diamond indicates the most significant SNP. c) Subcellular localization of Os01g41460 protein in rice protoplast cells. Cosubcellular localization of 35S::Os01g41460–GFP fusion protein with Mito marker (mitochondrial-mCherry) (top panels); cosubcellular localization of free GFP protein with Mito marker (bottom panels). Scale bars = 25 μm. d, e) Manhattan plot of GWAS analysis of mitochondrial SNP (HQ860792: 302370) in cucumber. The dashed horizontal line indicates the significance threshold. Only the SNPs exceeding this threshold were drawn in the zoomed-in plot, and the diamond indicates the most significant SNP. f) The loci that exhibit changes in editing efficiency between WT and transgenic lines. The changing tendencies are indicated by black arrows.

In rice, we identified a signal on chromosome 1 associated with 1 mitochondrial SNP (AB076665: 324769). The third most significant SNP was located in the 3′ untranslated region (UTR) region of gene *LOC_Os01g41610*, which encodes the g subunit of mitochondrial ATP synthase (Fig. 4a and b). As expected, in silico predictions from iPSORT, Predotar, and TargetP all indicate that the encoded protein is targeted to mitochondria (Emanuelsson et al. 2000; Bannai et al. 2002; Small et al. 2004). Moreover, we fused *LOC_Os01g41610* with green fluorescent protein (GFP) to verify its subcellular localization and confirmed that *LOC_Os01g41610* was exclusively targeted to mitochondria (Fig. 4c). It has been reported that the mitochondrial ATP synthase can improve the salt tolerance in seedling stage in rice (Zhang et al. 2006). Therefore, it is conceivable that mitonuclear interactions involving this candidate gene could be leveraged to enhance the adaptive capacity of rice under osmotic stresses.

In cucumber, we found that the gene *Csa5G601620*, which encodes a zinc finger RING/FYVE/PHD-type protein, was associated with a mitochondrial SNP (HQ860792: 302370). The most significant SNP located within *Csa5G601620* resulted in an amino acid change from Gly to Asp (Fig. 4d and e). Despite the prediction from 3 software tools indicating that *Csa5G601620* is targeted to the mitochondria, our microscopy assay showed that the encoded protein may localize to the plastids (supplementary fig. S6, Supplementary Material online). We also identified orthologs of *Csa5G601620* in the other 6 species (supplementary fig. S7, Supplementary Material online). These orthologs were not predicted to have mitochondrial targeting by software tools. Subsequent analysis revealed that none of these *Csa5G601620* orthologs exhibited mitonuclear association in the other species, potentially indicating divergence in mitonuclear interactions among different species. We also identified 10 genes with potential interactions with *Csa5G601620* by searching the STRING database (Szklarczyk et al. 2023; supplementary fig. S8, Supplementary Material online), 3 of which were predicted to exhibit mitochondrial targeting according to iPSORT (Bannai et al. 2002). Therefore, it is not clear whether this protein functions in the mitochondria, plastids, or both.

To investigate the phenotypic effects of *Csa5G601620* in cucumber, loss-of-function root mutants were obtained using CRISPR/Cas9 with a hairy root system induced by *Agrobacterium rhizogenes* (hereafter referred as Csa5G601620-CR) (supplementary fig. S9, Supplementary Material online). With respect to structure of the mitochondria, we found that both the wild type (WT) and Csa5G601620-CR exhibited regular shape, uniform size, and clear ridges, with no observable difference between them (supplementary fig. S10, Supplementary Material online). Next, we examined changes in RNA editing efficiency in the mitochondrial genome using transcriptomic data and identified 6 sites in coding sequence and 6 sites in introns exhibiting different RNA editing efficiencies between transgenic and WT lines, which were distributed across 5 mitochondrial genes (Fig. 4f). Moreover, we conducted an analysis of the distribution of these specific editing sites and observed that the majority of them is situated in 2 gene categories: *nad* (*nad2*, *nad4*, *nad7*) and *rps* (*rps4*, *rps10*). These genes encode components of the NADH dehydrogenase and small subunit of the mitochondrial ribosome, respectively. Finally, we examined the 6 specific editing sites within the coding sequence (CDS) region and observed that 4 are situated at the second position of the codon, while 2 are positioned at the third position of the codon, and none of them are located at the first position of the codon.

## Discussion

### Signatures and Potential Functional Consequences of Mitonuclear LD in Angiosperms

We generated variation maps of mitochondrial genomes for 7 selected species through a strict filtering strategy. The detected signals of association between mitochondrial and nuclear genomes in 7 species of angiosperms confirm that mitonuclear LD is common in these 7 species. As the only non-crop species in our study, arabidopsis displayed fewer signals compared with the 6 other domesticated species. We hypothesize that domestication could be a contributing factor to this observation, considering that cytonuclear interactions are anticipated to influence the domestication process (Wang, Li, et al. 2022). But more data and wider sampling should be included to test this hypothesis.

Mitonuclear interaction is vital to the adaptive evolution of eukaryotes (Lane 2011; Burton et al. 2013; Sloan et al. 2017). Signals of mitonuclear coevolution and fitness effects have been detected in different taxa such as insects, mammals, fungi, and plants (Barreto et al. 2018; Weaver et al. 2022; Biot-Pelletier et al. 2023). For example, mitonuclear differentiation in birds may have been associated with local adaptation, mixing of different mitochondrial haplotypes. Likewise, nuclear genomes in African hybrid cattle may have been a source of phenotypic diversity, and a fertility restorer gene was identified based on mitonuclear incompatibility in *Citrus* (Wang et al. 2021; Wang, Li, et al. 2022; Ward et al. 2022). Moreover, the association between mitochondrial variations and nuclear genome variation has been investigated in relation to some human diseases (West et al. 2005; Sloan et al. 2015; Ludwig-Slomczynska et al. 2020).

Through GO enrichment analyses and additional functional experimentation, we found some evidence to support the hypothesis that mitonuclear LD in plants preferentially involves nuclear genes with mitochondrial functions. Moreover, we also observed that associated genes exhibited higher nucleotide polymorphism, which can be partially explained by the fact that a region with greater nuclear diversity provides more statistical power to detect associations (Mills and Rahal 2020). This phenomenon is somewhat analogous to the observation in GWAS analyses, where signals often manifest in SNP hotspot regions (Long and Xue 2021). Given that the majority of *Rf* genes belongs to the PPR gene family (Chase 2007), the numerous PPR genes identified in our study could serve as high-priority candidates for further analysis as potential *Rf* genes. Interestingly, our subcellular localization assays identified a gene exhibiting mitonuclear LD (*Csa5G601620* in the “zinc finger RING/FYVE/PHD-type” gene family in cucumber) that might not be targeted to mitochondria but nonetheless had an effect on RNA editing efficiency of mitochondrial genes when knocked out. Although such functional consequences could be indirect, they raise the possibility that nuclear genes may be involved in mitonuclear coevolution and mitochondrial function even if the proteins that they encode are not transported into mitochondria.

It is also important to consider that there could be other evolutionary driving forces besides selection on epistatic mitonuclear interaction that could be involved in establishing the observed associations. For example, LD between unlinked loci (e.g. nuclear and mitochondrial genes) can also arise from genetic drift and population subdivision/stratification (Slatkin 2008; Schaid et al. 2018). In addition, selection could act independently to increase the frequencies of alleles at 2 different loci even if their fitness effects do not interact epistatically. Although our GWAS approach attempts to account for population stratification (see Materials and Methods), it is possible that such factors still contribute to observed associations.

The mitochondrial SNPs involved in the associations highlighted in Fig. 4 are distinctive in that the alternative alleles at each of the 2 loci are exclusively found in heteroplasmic individuals, i.e. none of the sampled individuals had 100% frequency for these alternative alleles (supplementary fig. S11, Supplementary Material online). This pattern of exclusive heteroplasmy stands in contrast to other identified mitochondrial SNPs in our analysis, for which both alleles can be found in a homoplasmic state in at least some individuals. The mechanisms that could create a statistical correlation between nuclear genotype in specific regions of the genome and allele frequencies for mitochondrial heteroplasmies are not clear, but such observations parallel recent findings in humans (Gupta et al. 2023). Our analysis pipeline included steps to filter spurious variants that could arise from mapping artifact-associated repeats in the mitochondrial genome or insertions of mitochondrial DNA into the nucleus (numts). However, we cannot rule out the possibility that variants exclusively found as heteroplasmies could result from repeats or numts that are specific to a subset of sampled individuals and not present in the reference genomes used for read mapping. Long-read sequencing of each individual could be used to investigate such possibilities in more detail in the future.

### Advantages and Limitations of GWAS for Investigation of Mitonuclear LD

Due to the interest in mitonuclear coevolution in eukaryotes, many methods have been developed to investigate associations between organelle and nuclear genomes, and a large number of experiments have been conducted to verify the functional consequences of these associations. For instance, a popular and convenient method is to predict the subcellular localization based the characteristics of the N-terminal signal peptide by various machine learning algorithms, but this method cannot predict involvement in specific enzyme complexes or functional pathways (Emanuelsson et al. 2000; Small et al. 2004; Fukasawa et al. 2015; Almagro Armenteros et al. 2019). Analyses of gene coexpression or protein–protein interactions can also identify functional relationships between genes (Obayashi et al. 2009; Xing et al. 2016). ERC is another prediction method for proteins that are functionally related and/or coevolving by calculating genetic distances or branch lengths of gene trees from 2 potentially interacting genes (Clark et al. 2012; Yan et al. 2019; Forsythe et al. 2021). ERC is designed for detecting associations on long evolutionary (i.e. phylogenetic) timescales. More detailed wet-lab investigations and functional experimentation are generally necessary to verify functional cytonuclear interactions, but these approaches are often laborious with low throughput and the efficiency (Schafer et al. 2020; Cao et al. 2022; Wang, Blondel, et al. 2022). Hence, existing methods have their own strengths and weaknesses with respect to identifying “mismatched” combinations of nuclear and cytoplasmic variations within species.

In this study, we used mitochondrial variants as “phenotypes” in a GWAS-like analysis to find relationships with nuclear polymorphisms within species at genome-wide level. We successfully detected signals of mitonuclear association in 7 species across angiosperms. Similar methods have also been applied to identify mitonuclear interaction signals in humans, birds and cattle, etc. (Hodgkinson et al. 2014; Wang et al. 2021; Ward et al. 2022). Thus, GWAS can successfully identify nuclear genes associated with organelle variants, even those with low allele frequencies. Regions of interest include nuclear genes with unknown functions, which may contribute to finding the underlying mechanisms of CMS and diseases (Alonso-Blanco et al. 2016; Tam et al. 2019). On the other hand, the identified nuclear genes likely account for only a small part of this complex genetic variation, which will depend on setting sensitivity thresholds, population size, etc. (Manolio et al. 2009; Yang et al. 2011). Furthermore, candidates identified with GWAS signals are not necessarily causal variants and genes. Additional tests are required to assess functional effects (McCarthy et al. 2008). Thus, GWAS of mitonuclear LD is a potential method for identifying mitonuclear interactions, but more efforts are still needed to assess its sensitivity and accuracy.

## Materials and Methods

### Mitochondrial Variants Calling and Filtering Using WGS Data

We used BWA-mem (v6.0.2) (Li and Durbin 2009) with default parameters to map the WGS data against the mitochondrial genome. Subsequently, the SAM file was filtered to retain only the uniquely mapped reads using SAMtools (v0.1.18) (Wysoker et al. 2009). Next, duplicates were marked using picard (v1.119) (http://broadinstitute.github.io/picard/). Finally, we created an index for all the resulting BAM files for subsequent analysis using GATK (v4.1.3.0) (McKenna et al. 2010).

After obtaining the index file, we utilized the Mutect2 package in GATK (v4.1.3.0) to identify variations in mitochondrial mode. The command line used was as follows: “gatk Mutect2 --java-options ‘-Xmx20G -D java.io. tmpdir=./’ --native-pair-hmm-threads 2 --mitochondria-mode true -R mitochondrial.sequence.fa -O result.vcf -I S1.new.sorted.bam -I S2.new.sorted.bam -I SN.new.sorted.bam.” To reduce the risk of false positive variants, we applied the FilterMutectCalls package in GATK (v4.1.3.0) with the following command: “gatk FilterMutectCalls --mitochondria-mode true -R mitochondrial.sequence.fa -V result.vcf -O filtered.result.vcf.” To further ensure the validity of the variant calls, only biallelic sites missing genotype calls from ≤40% of individuals were retained for further analysis. To eliminate potential false positives resulting from multiple copies (supplementary fig. 12a, Supplementary Material online), we calculated the depth of coverage for each mitochondrial variant and the average depth value of the entire mitochondrial genome. Sites with depth values larger than 1.5 times the average depth were discarded. In addition, mitochondrial SNPs covered by fewer than 4 reads were filtered. We also used BLAST (v2.5.0+) to exclude loci located in repeated regions (≥100 bp) within the reference mitochondrial genome (supplementary fig. 12b, Supplementary Material online; Camacho et al. 2009). Finally, we extracted and excluded regions with homology (≥100 bp) between the mitochondrial genome and nuclear or plastid genomes using mummer (v3.23) (Marcais et al. 2018).

### GWAS

1. We first extracted depth values from the vcf file that had been filtered as described above. Next, we computed the reference allele frequency by dividing the number of reads supporting the reference genome by the total number of reads, and we refer to this value as the “phenotype.” Missing loci in specific samples were labeled “NA.”
2. All nuclear SNPs were obtained from online sources, and all these nuclear genomic variations were identified using rigorous pipelines, and their reliability has been validated in previous research papers, which we have cited. After identifying these nuclear genomic variations, we implemented the filtering with criteria set at missing > 40% and MAF < 0.05 (supplementary table S5, Supplementary Material online).
3. Integrating the nuclear genome SNPs and the phenotyping data from the previous step, we performed GWAS using the efficient mixed model association expedited (EMMAX) program (Kang et al. 2010). Population stratification and hidden relatedness were modeled with a kinship (K) matrix in the emmax-kin-intel package of EMMAX.
4. We applied the threshold value of −log_10_(*P*) > 6 to identify the candidate-associated regions (the threshold value of 6 is larger than the value −log_10_(0.05/*n*), where *n* is the effective number of independent SNPs, in most species in the study). The R package qqman (Turner 2018) was used to draw Manhattan plots.
5. The zoomed-in plots of GWAS signals were drawn by LDBlockShow (v1.36) (Dong et al. 2021). Association “signals” refer to clusters of SNPs that surpass the threshold on the Manhattan plots, defined as the range between the left- and right-most SNPs beyond the aforementioned threshold.
6. We also repeated our analysis after transforming the reference allele frequency from a continuous variable into binary trait (0 indicates reference allele frequency < 0.05, while 1 indicates reference allele frequency > 0.95). This was performed in arabidopsis and cucumber for a sample of 2 and 4 mitochondrial variants, respectively (supplementary fig. S13, Supplementary Material online).
7. The most significant SNP and the corresponding gene within an associated region were considered first, as they represent the most likely candidate genes within a given peak.

### Calculation and Analysis of Ka/Ks

To calculate Ka/Ks, we first generated a consensus sequence for each species by substituting the reference genotype with the most common genotype to avoid reference bias. This consensus sequence was then compared against the sequence from an outgroup species (supplementary fig. S14, Supplementary Material online) and used to calculate Ka/Ks with TBtools (v1.120) (Chen et al. 2020). The choice of outgroup species for each focal species was as follows: rice (focal species): OB-rice (outgroup species) (Shang et al. 2022), tomato (focal species): potato (outgroup species) (Li et al. 2023), maize (focal species): sorghum (outgroup species) (Hufford et al. 2021), cucumber (focal species): melon (outgroup species) (Zhao et al. 2019), watermelon (focal species): cucumber (outgroup species) (Guo et al. 2013), and melon (focal species): cucumber (outgroup species) (Zhao et al. 2019).

### Identification of Orthogroups and GO Enrichment Analysis

All the associated genes were input into OrthoFinder2 (v2.3.8) (Emms and Kelly 2019) using the command “orthofinder -f input_directory -t 5” to identify orthogroups. The functional information for the chosen orthogroups was extracted manually by referring to the gene annotation information within each orthogroup. We conducted GO enrichment analysis using TBtools software and GO term information derived from whole gene annotation as the background.

### The Analysis of Nuclear Diversity (π)

π was then calculated using Vcftools (v0.1.16) software with the command “vcftools --vcf output.vcf --window-pi 100000 --window-pi-step 10000 --out pi.result.”

### Identification of RNA Editing Sites

To identify RNA editing sites, transformed roots with GFP fluorescence (see below) were collected ∼1.5 cm long piece of root tissue below the base of the hypocotyl. Hairy roots transformed with empty pBSE402 vector were used as negative control. Each group consists of 3 biological replications (8 hairy roots per replicate). Total RNA was extracted with TRIzol reagent (Invitrogen, USA); libraries were constructed according to the manufacturer’s instructions (Novogene) and then sequenced with Illumina HiSeq 4000 sequencing platform. We aligned the clean reads to the mitochondrial genome using HISAT2 (v2.2.1; Kim et al. 2015). Reads with mapping quality lower than 30 were filtered with SAMtools (v0.1.18). Picard (v1.119) (http://broadinstitute.github.io/picard/) was used to mark duplicates. The HaplotypeCaller tool in GATK (v4.1.3.0) was used to call SNPs. To avoid false positives in RNA editing site calling, we excluded any SNPs that were also detected in WGS data. We also only included SNPs that were detected in all 3 biological replicates. Finally, we excluded any variants that were not C-to-T substitutions on the transcribed strand.

### *A. rhizogenes*-Mediated Hairy Root Transgenic System in Cucumber

To generate a CRISPR/Cas9 based genome-editing vector, guide RNAs for *Csa5G601620* were designed with CRISPER-GE (http://skl.scau.edu.cn/home/) and then cloned into binary vector pBSE402 using the Golden Gate assembly method. These constructed vectors were transformed into *A. rhizogenes* strain Ar. Qual (CAT#: AC1040M). Primer sequences are listed in supplementary table S7, Supplementary Material online.

Cucumber Cu2 peeled seeds were sterilized by 75% (*v*/*v*) ethanol for 30 s and 0.3% (*v*/*v*) sodium hypochlorite solution for 15 min and then washed with sterilized water 3 to 5 times. After removing excess water, the sterilized seeds were germinated on Murashige and Skoog (MS) medium at 25 °C for 2 d in darkness and then transferred to 25 °C for ∼5 d under a 16/8 h light/dark photoperiod until cotyledons fully expanded. Cotyledons were cut off at the basal and tip end and soaked in transformed *A. rhizogenes* strain (OD_600_ = 0.2) at 28 °C for 20 min and then cocultivated on MS solid medium for 2 d at 23 °C in darkness. To regenerate roots, explants were transferred to MS solid medium (with 200 mg/L timentin) for ∼2 wks at 25 °C under a 16/8 h light/dark photoperiod.

### Subcellular Localization

The coding sequence (without stop codon) of *Csa5G601620* was cloned from “cu2” cDNA (primers are listed in supplementary table S6, Supplementary Material online) and inserted into 131-35S-YFP vector (41) with In-Fusion method to generate *35S:Csa5G601620-YFP* and then transformed into *Agrobacterium tumefaciens* strain GV3101. The recombinant expression vector was mixed with P19 and then injected into tobacco leaves, with *35S:YFP* and P19 injection as control. The injected tobacco plants were kept in darkness for 12 h and then cultured under light conditions for 3 d before leaf samples were observed under laser scanning confocal microscope (Leica TCS SP8). Plastids were identified by their typical red chlorophyll autofluorescence.

The coding sequence (without stop codon) of *LOC_Os01g41610* was cloned from Nipponbare cDNA (primers are listed in supplementary table S6, Supplementary Material online) and inserted into pBI221-GFP vector (42) with In-Fusion method to generate *35S:LOC_Os01g41610-GFP*. The recombinant expression vector was mixed with mitochondrial-mCherry (43) and transformed into protoplasts derived from rice leaf sheaths by polyethylene glycol (PEG)-mediated DNA transformation (44), with *35S:GFP* and mitochondrial-mCherry as a control. The transformed protoplasts were incubated for 12 h in 27 °C and observed under laser scanning confocal microscope.

## Supplementary Material

msae028_Supplementary_Data

## Data Availability

All the data used in this study were publicly accessible (accessions listed in supplementary table S2, Supplementary Material online): Arabidopsis, SRP056687; rice, PRJEB6180; maize, PRJNA399729; tomato, SRP045767; melon, PRJNA565104; cucumber, SRA056480; and watermelon, SRP188834 and SRP192188.
