# Supplementary material for: Association Analysis Provides Insights into Plant Mitonuclear Interactions
Source: Mol Biol Evol. 2024 Feb 7;41(2):msae028. doi: 10.1093/molbev/msae028 (PMC10875325; doi:10.1093/molbev/msae028)
Supplement: msae028_Supplementary_Data [file msae028_supplementary_data.zip › Supplementary Figure 9.pdf]

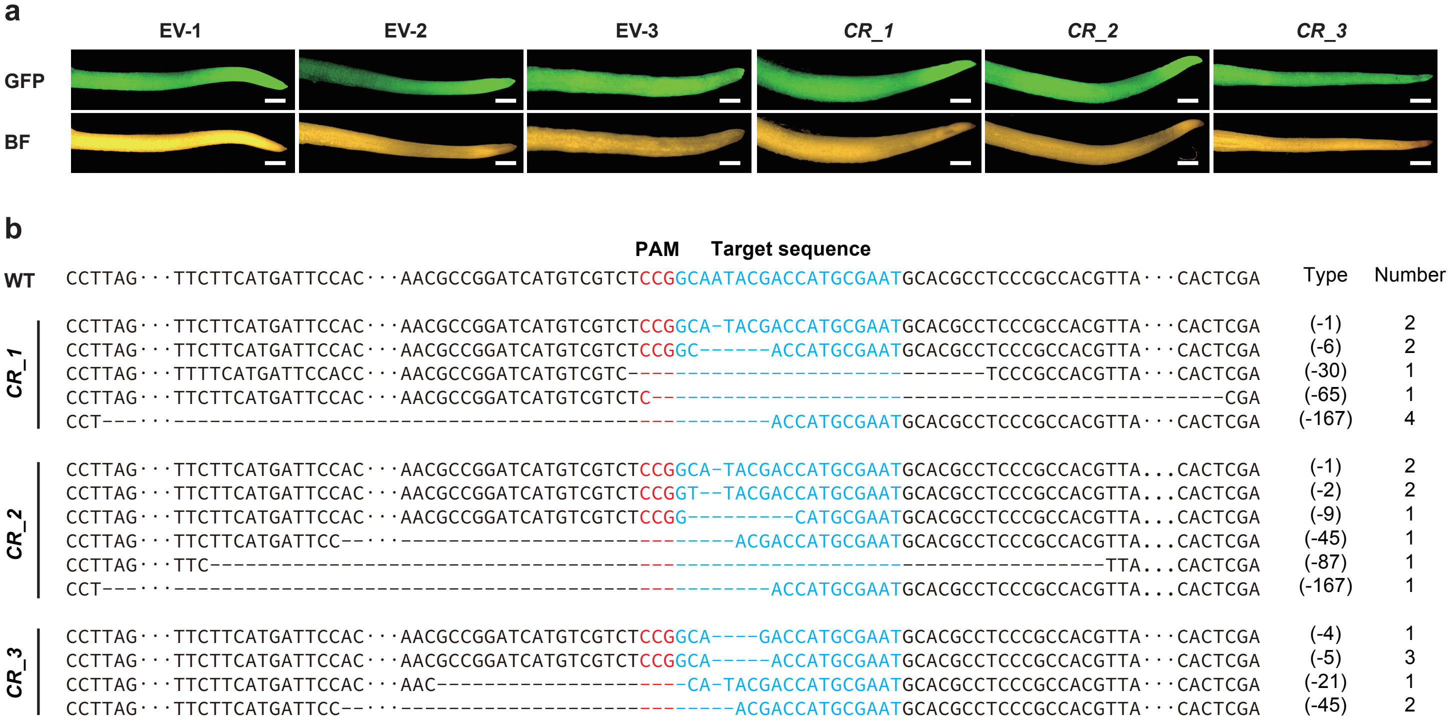
a
EV-1
EV-2
EV-3
CR_1
CR_2
CR_3
GFP
BF
b
PAM
Target sequence
WT CCTTAG···TTCTTCATGATTCCAC···AACGCCGGATCATGTCGTCTCCGGCAATACGACCATGCGAATGCACGCCTCCCGCCACGTTA···CACTCGA
Type
Number
CR_1
CCTTAG···TTCTTCATGATTCCAC···AACGCCGGATCATGTCGTCTCCGGCA-TACGACCATGCGAATGCACGCCTCCCGCCACGTTA···CACTCGA (-1) 2
CCTTAG···TTCTTCATGATTCCAC···AACGCCGGATCATGTCGTCTCCGGC------ACCATGCGAATGCACGCCTCCCGCCACGTTA···CACTCGA (-6) 2
CCTTAG···TTTTCATGATTCCACC···AACGCCGGATCATGTCGTC------------------------------TCCCGCCACGTTA···CACTCGA (-30) 1
CCTTAG···TTCTTCATGATTCCAC···AACGCCGGATCATGTCGTCTC--CGA (-65) 1
CCT---···--ACCATGCGAATGCACGCCTCCCGCCACGTTA···CACTCGA (-167) 4
CR_2
CCTTAG···TTCTTCATGATTCCAC···AACGCCGGATCATGTCGTCTCCGGCA-TACGACCATGCGAATGCACGCCTCCCGCCACGTTA...CACTCGA (-1) 2
CCTTAG···TTCTTCATGATTCCAC···AACGCCGGATCATGTCGTCTCCGGT--TACGACCATGCGAATGCACGCCTCCCGCCACGTTA...CACTCGA (-2) 2
CCTTAG···TTCTTCATGATTCCAC···AACGCCGGATCATGTCGTCTCCGG---------CATGCGAATGCACGCCTCCCGCCACGTTA...CACTCGA (-9) 1
CCTTAG···TTCTTCATGATTCC--···----------------------------ACGACCATGCGAATGCACGCCTCCCGCCACGTTA...CACTCGA (-45) 1
CCTTAG···TTC---TTA...CACTCGA (-87) 1
CCT---···--ACCATGCGAATGCACGCCTCCCGCCACGTTA...CACTCGA (-167) 1
CR_3
CCTTAG···TTCTTCATGATTCCAC···AACGCCGGATCATGTCGTCTCCGGCA----GACCATGCGAATGCACGCCTCCCGCCACGTTA···CACTCGA (-4) 1
CCTTAG···TTCTTCATGATTCCAC···AACGCCGGATCATGTCGTCTCCGGCA-----ACCATGCGAATGCACGCCTCCCGCCACGTTA···CACTCGA (-5) 3
CCTTAG···TTCTTCATGATTCCAC···AAC---------------------CA-TACGACCATGCGAATGCACGCCTCCCGCCACGTTA···CACTCGA (-21) 1
CCTTAG···TTCTTCATGATTCC--···----------------------------ACGACCATGCGAATGCACGCCTCCCGCCACGTTA···CACTCGA (-45) 2
