# Supplementary material for: Association Analysis Provides Insights into Plant Mitonuclear Interactions
Source: Mol Biol Evol. 2024 Feb 7;41(2):msae028. doi: 10.1093/molbev/msae028 (PMC10875325; doi:10.1093/molbev/msae028)
Supplement: msae028_Supplementary_Data [file msae028_supplementary_data.zip › Supplementary Tables.pdf]

Supplementary Table 1 **S**ummary of mitochondrial (mt) variation maps.

Species	Mt-genome Size	Total mt-mutations	Intergenic mt-mutations	Genic mt-mutations	Genic Rate	Average depth (×)
Arabidopsis	365kb	742	541	201	27.09%	98.88
Rice	486kb	624	500	124	19.87%	113.96
Maize	565kb	824	587	237	28.76%	127.91
Tomato	446kb	2,888	2,428	460	15.93%	120.33
Watermelon	376kb	1,013	789	224	22.11%	196.87
Cucumber	1.7Mb	5,364	4,756	608	11.33%	130.28
Melon	2.9Mb	17,578	16,718	860	4.89%	105.04

Supplementary Table 4 GO enrichment analysis

Species	GO terms	Class	Description	P-value
Melon	GO:1990542	Biological Process	mitochondrial transmembrane transport	0.0058
	GO:0006123	Biological Process	mitochondrial electron transport, cytochrome c to oxygen	0.0066
	GO:0005751	Cellular Component	mitochondrial respiratory chain complex IV	0.0068
	GO:0045041	Biological Process	protein import into mitochondrial intermembrane space	0.0127
	GO:0007005	Biological Process	mitochondrion organization	0.0158
	GO:0006626	Biological Process	protein targeting to mitochondrion	0.0219
	GO:0070585	Biological Process	protein localization to mitochondrion	0.0219
	GO:0000002	Biological Process	mitochondrial genome maintenance	0.0299
	GO:0098573	Cellular Component	intrinsic component of mitochondrial membrane	0.05
Tomato	GO:0031314	Cellular Component	extrinsic component of mitochondrial inner membrane	0.0003
Watermelon	GO:0090615	Biological Process	mitochondrial mRNA processing	0.0445
Cucumber	GO:0000959	Biological Process	mitochondrial RNA metabolic process	0.0392

(*Continued*)

Species	GO terms	Class	Description	P-value
Maize	GO:0005747	Cellular Component	mitochondrial respiratory chain complex I	0.0032
	GO:0005746	Cellular Component	mitochondrial respiratory chain	0.0057
	GO:0098800	Cellular Component	inner mitochondrial membrane protein complex	0.0067
	GO:0006850	Biological Process	mitochondrial pyruvate transport	0.008
	GO:0005761	Cellular Component	mitochondrial ribosome	0.0118
	GO:0044455	Cellular Component	mitochondrial membrane part	0.021
	GO:0090615	Biological Process	mitochondrial mRNA processing	0.0249
	GO:0097250	Biological Process	mitochondrial respiratory chain supercomplex assembly	0.0249
	GO:0098798	Cellular Component	mitochondrial protein complex	0.0259
	GO:0000275	Cellular Component	mitochondrial proton-transporting ATP synthase complex	0.0338
	GO:0000963	Biological Process	mitochondrial RNA processing	0.0359
	GO:0006264	Biological Process	mitochondrial DNA replication	0.0359
	GO:0005762	Cellular Component	mitochondrial large ribosomal subunit	0.0439

Supplementary Table 5 Summary of nuclear SNPs.

Species	Number of nuclear SNPs
Arobidopsis	1,124,355
Cucumber	1,854,303
Maize	17,503,471
Melon	3,981,154
Rice	2,838,155
Tomato	2,990,613
Watermelon	3,259,969

Supplementary Table 6 Primer sequences used in subcellular localization.

Csa5G601620 - Primers sequence
Csa5G601620-F: gaggatctcgagcggggtaccATGATTCCACGATCAAAAGAT
Csa5G601620-R: agcggccgctgtacaggtaccCCAAGCTTGAACAATTCGATC
LOC_Os01g41610- Primers sequence
LOC_Os01g41610-F：gaggatctcgagcggggtaccATGGCGTCGAGGCTGGCGC
LOC_Os01g41610-R：agcggccgctgtacaggtaccGACCTTATAGCCGGTTATGG

Supplementary Table 7 sgRNA and primer sequences used in hairy root transgenic system.

Csa5G601620-sgRNA sequence
Csa5G601620-sgRNA-F: ATTGATTCGCATGGTCGTATTGC
Csa5G601620-sgRNA-R: AAACGCAATACGACCATGCGAAT
Csa5G601620-Primers sequence
Csa5G601620-test-F：ATTTATATGAAAAGTCGGCCTCAG
Csa5G601620-test-R：ATAAACAAACGAGCACCTTCTAGG
