# Supplementary figures and images for: Association Analysis Provides Insights into Plant Mitonuclear Interactions
Source: Mol Biol Evol. 2024 Feb 7;41(2):msae028. doi: 10.1093/molbev/msae028 (PMC10875325; doi:10.1093/molbev/msae028)

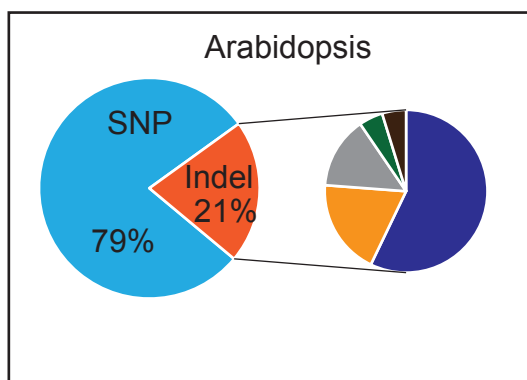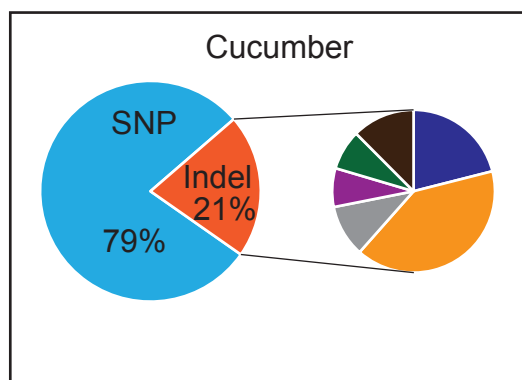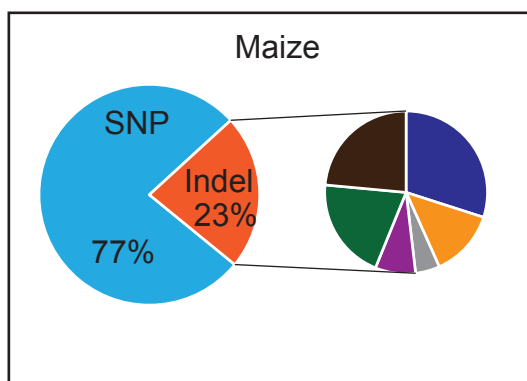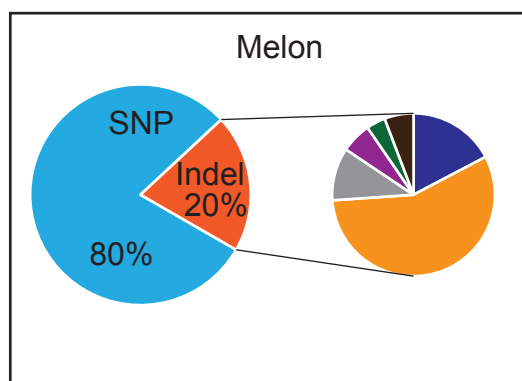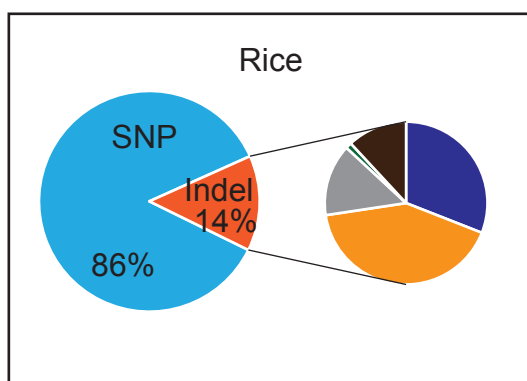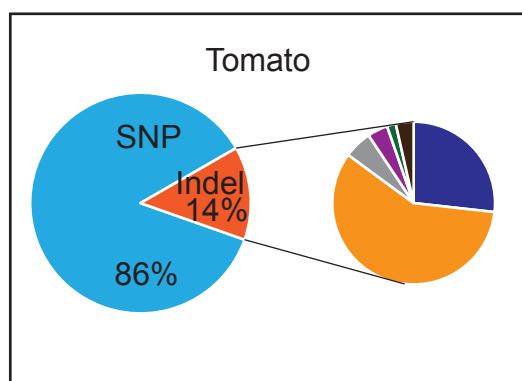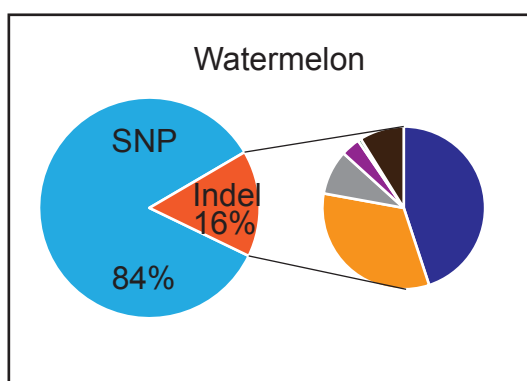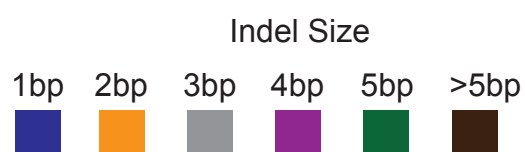

Supplement: msae028_Supplementary_Data [file msae028_supplementary_data.zip › Supplementary Figure 1.pdf]

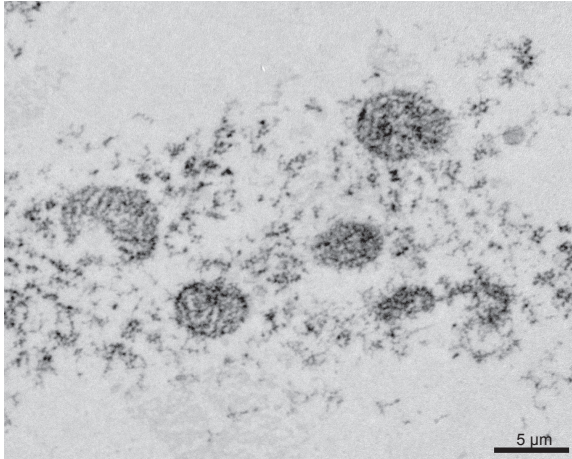

**WT**

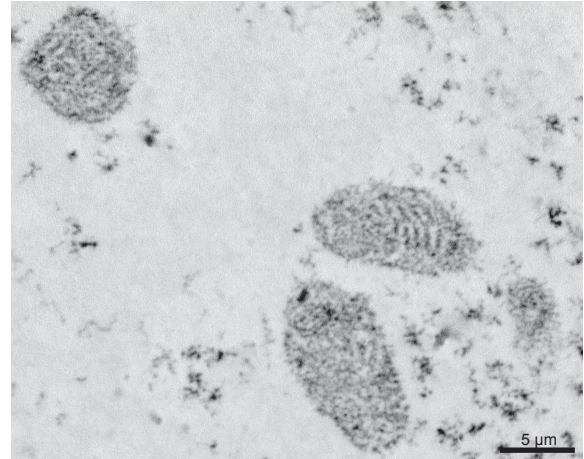

***Csa5G601620-CR***

Supplement: msae028_Supplementary_Data [file msae028_supplementary_data.zip › Supplementary Figure 10.pdf]

a

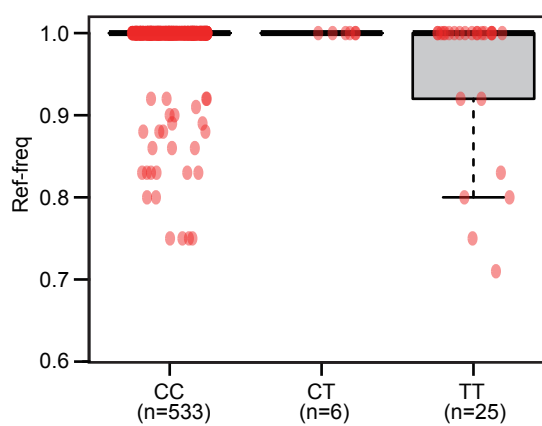

b

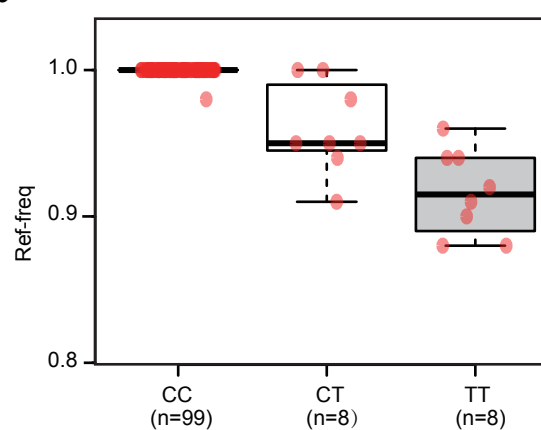

Supplement: msae028_Supplementary_Data [file msae028_supplementary_data.zip › Supplementary Figure 11.pdf]

a

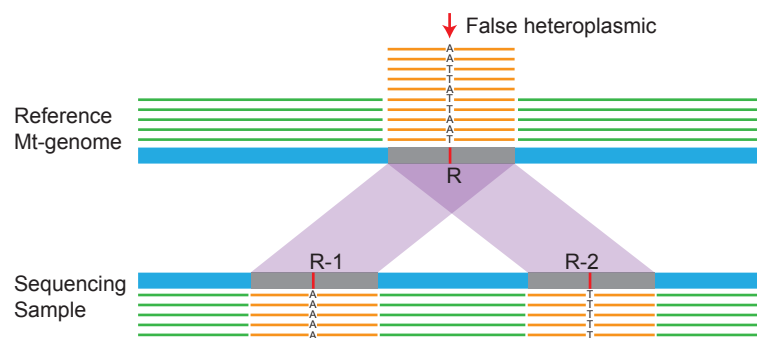

b

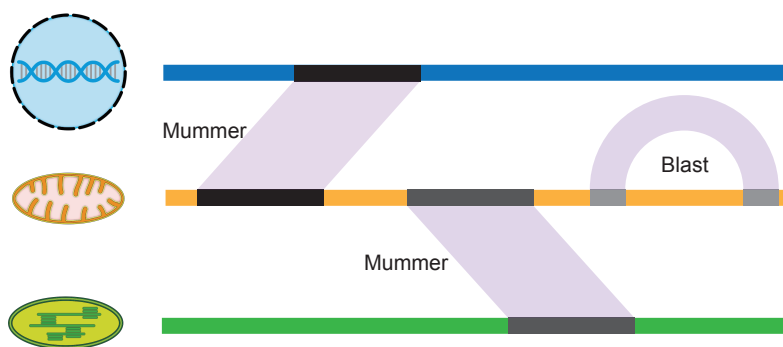

Supplement: msae028_Supplementary_Data [file msae028_supplementary_data.zip › Supplementary Figure 12.pdf]

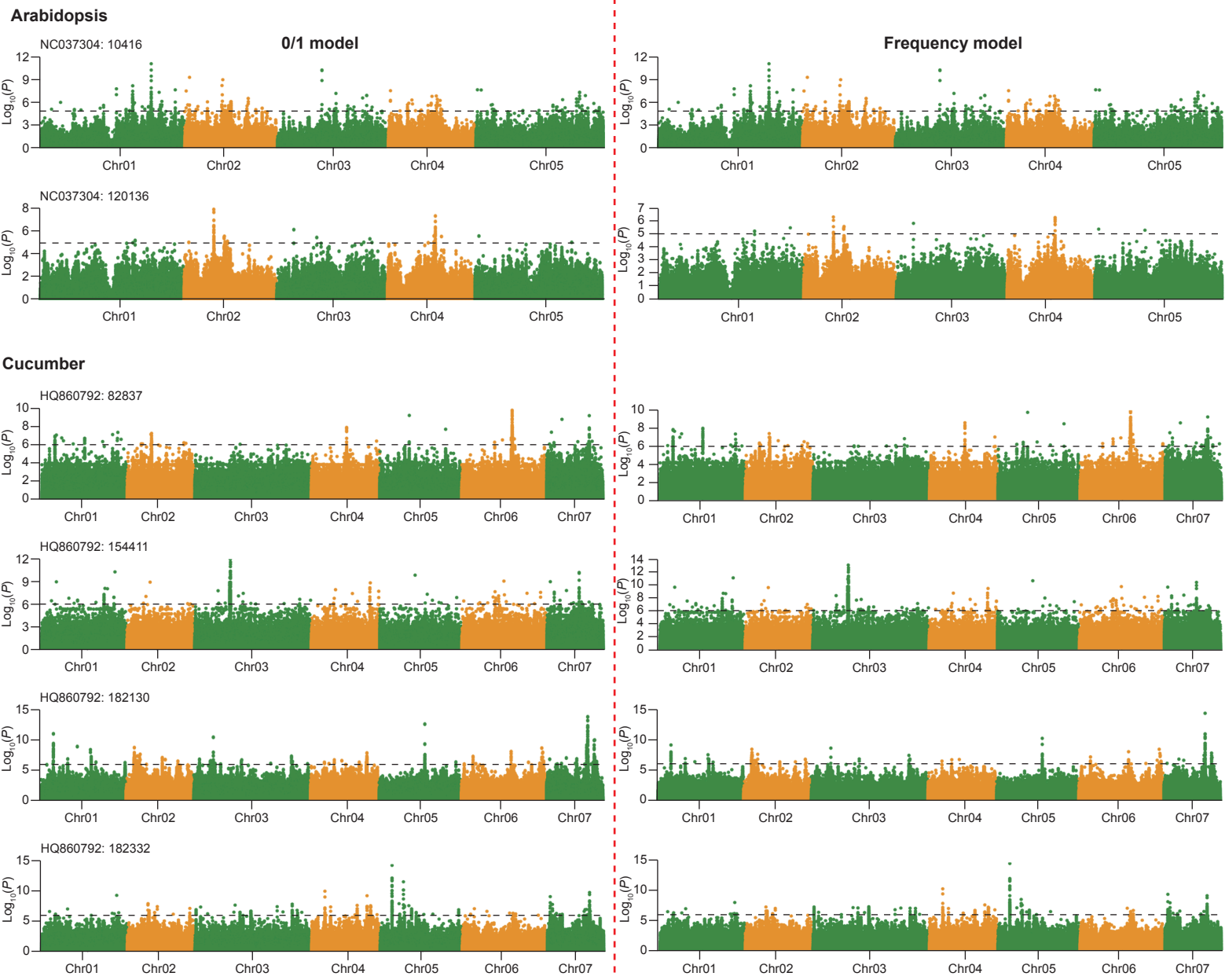

Supplement: msae028_Supplementary_Data [file msae028_supplementary_data.zip › Supplementary Figure 13.pdf]

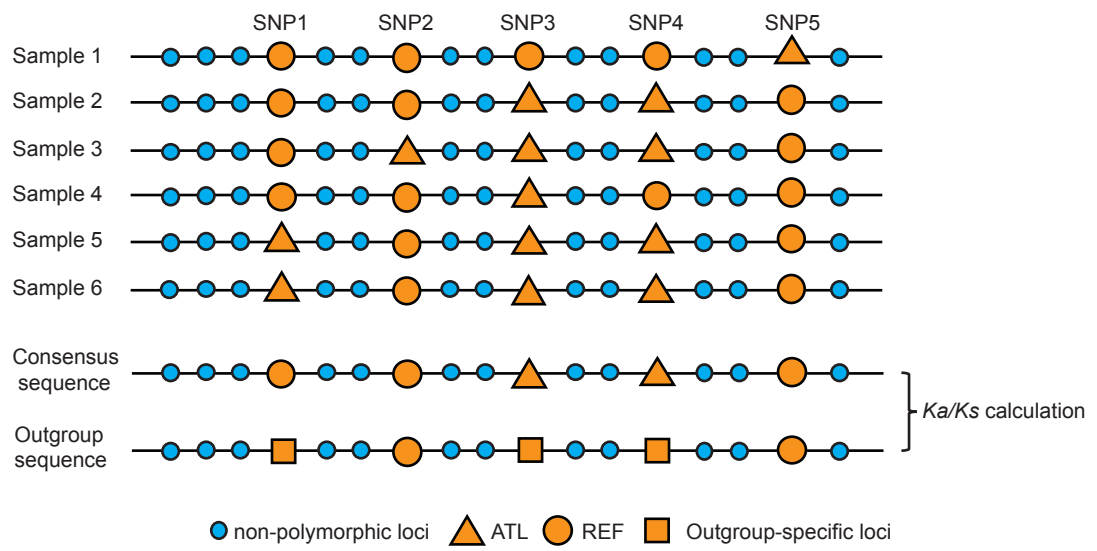

Supplement: msae028_Supplementary_Data [file msae028_supplementary_data.zip › Supplementary Figure 14.pdf]

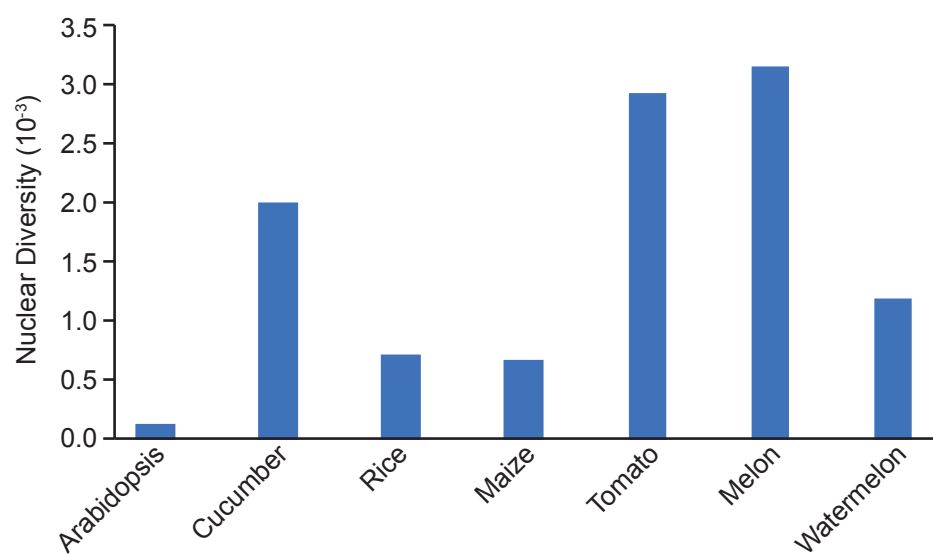

Supplement: msae028_Supplementary_Data [file msae028_supplementary_data.zip › Supplementary Figure 2.pdf]

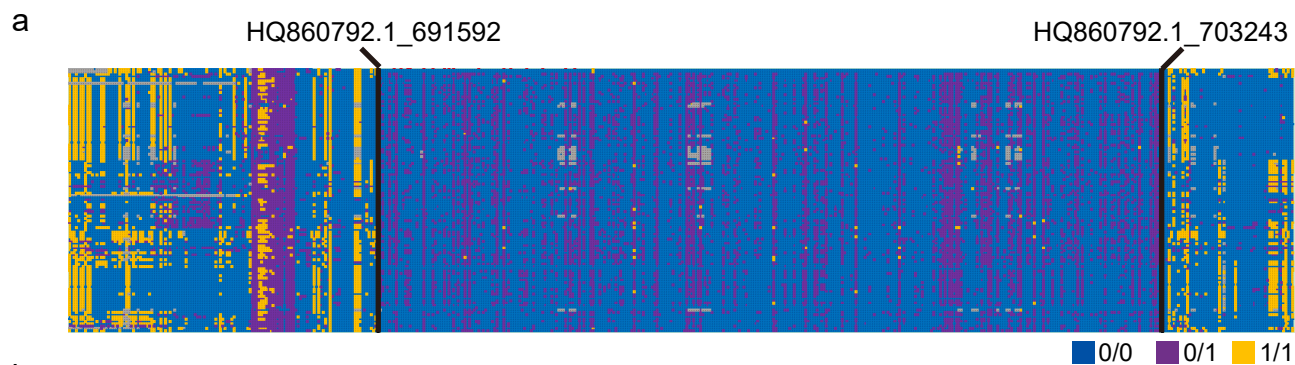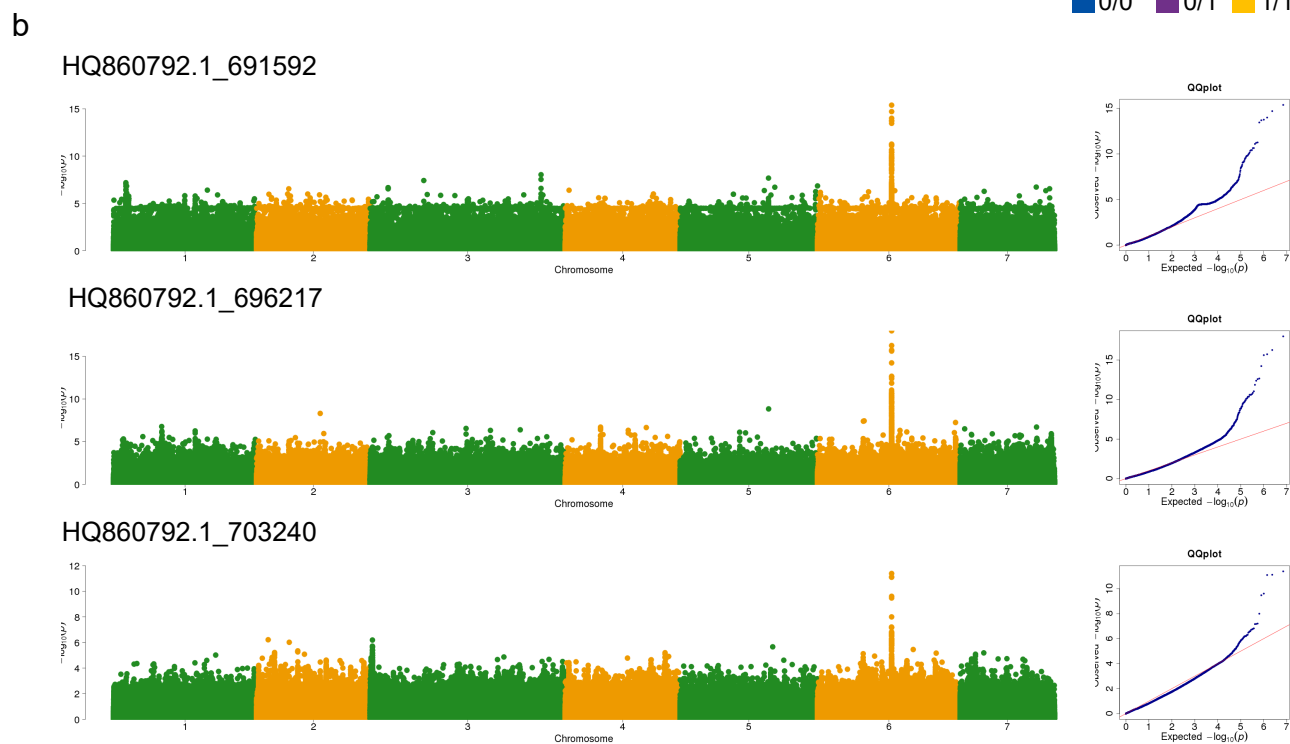

Supplement: msae028_Supplementary_Data [file msae028_supplementary_data.zip › Supplementary Figure 3.pdf]

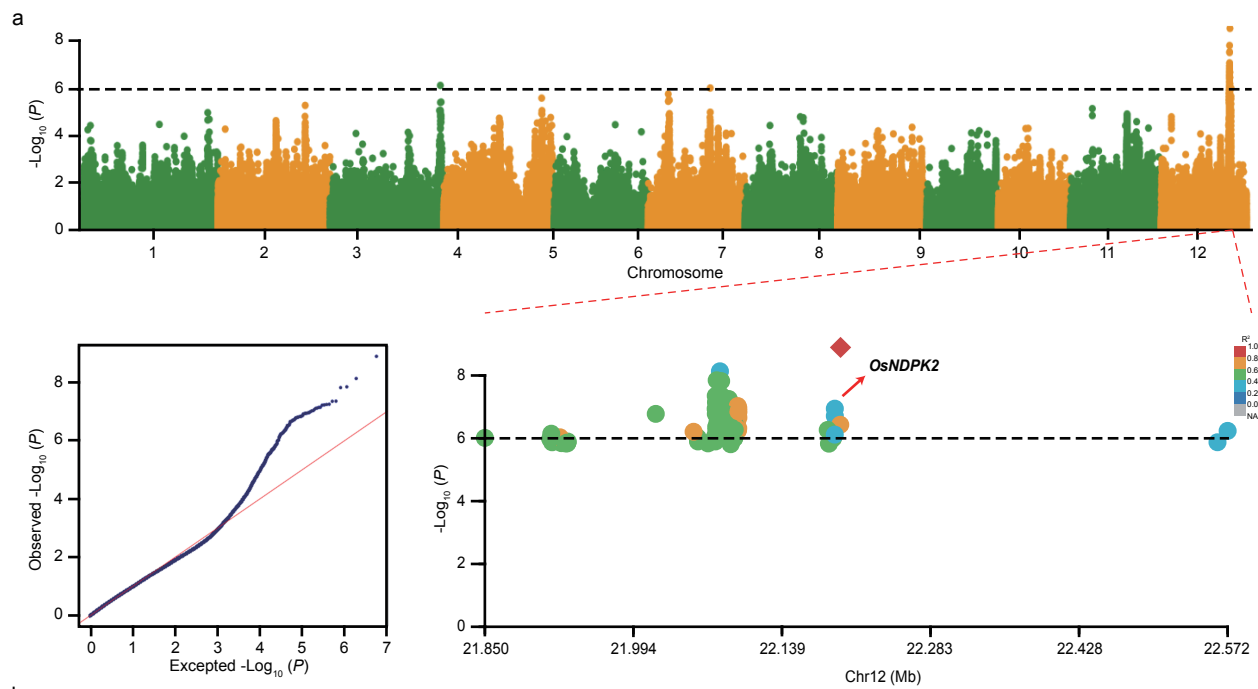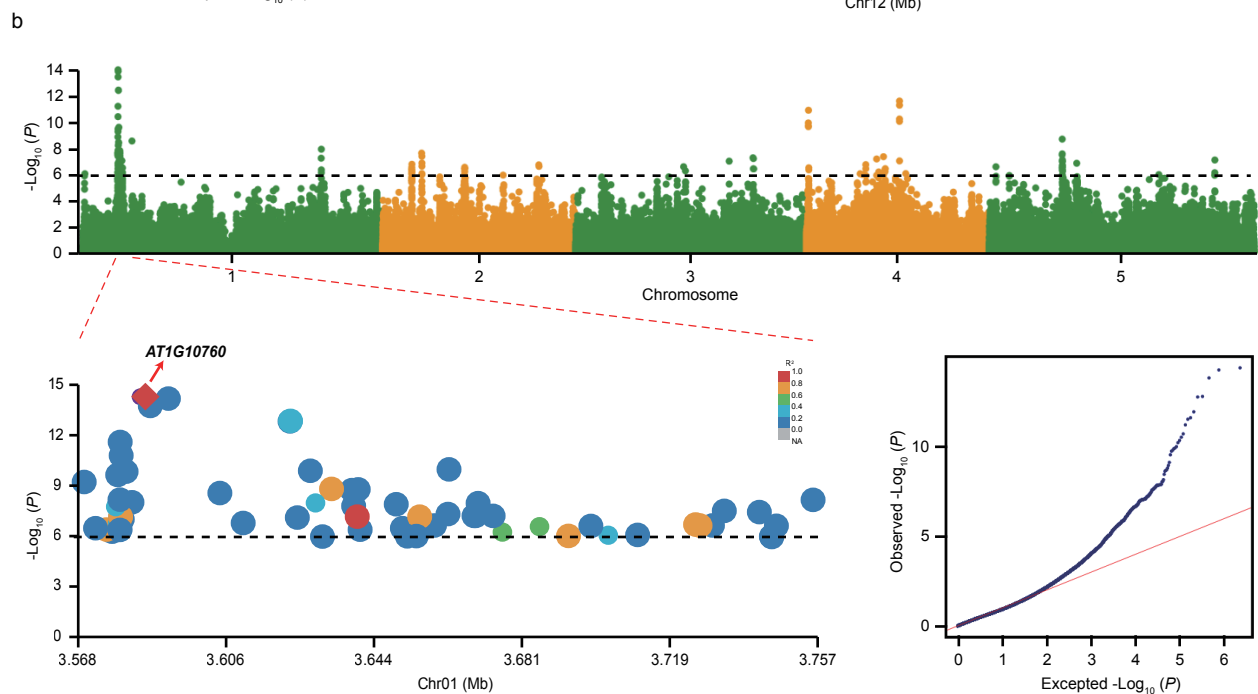

Supplement: msae028_Supplementary_Data [file msae028_supplementary_data.zip › Supplementary Figure 4.pdf]

AY506529.1\_342559

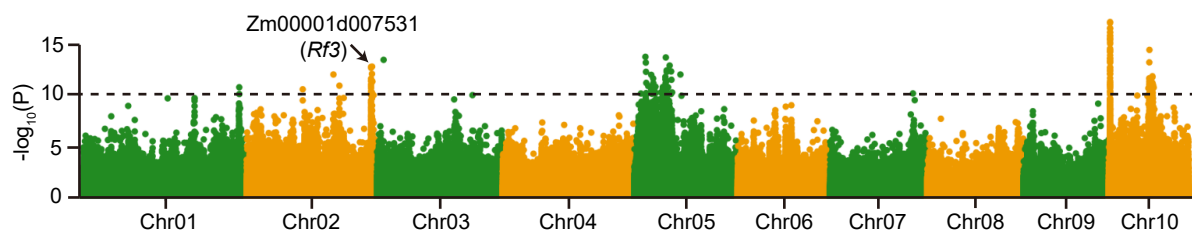

Supplement: msae028_Supplementary_Data [file msae028_supplementary_data.zip › Supplementary Figure 5.pdf]

YFP

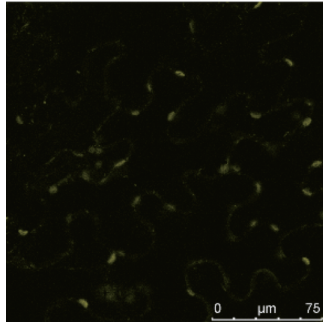

mCherry

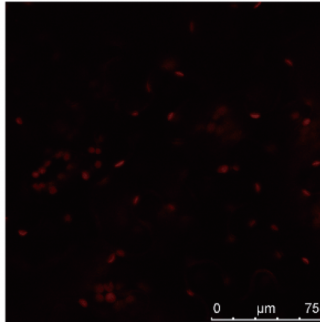

Bright field

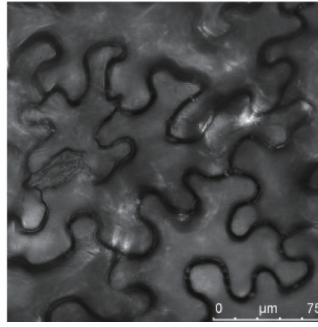

Merge

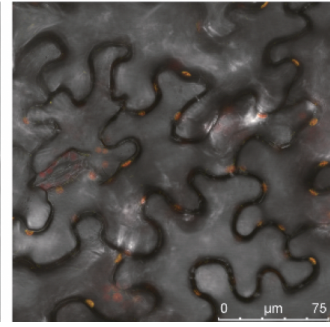

*Csa5G601620*-YFP  
+Auto chl

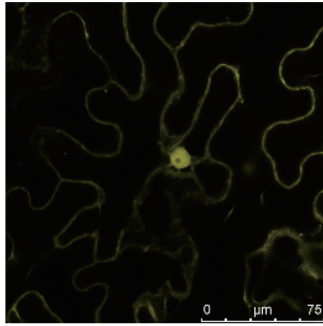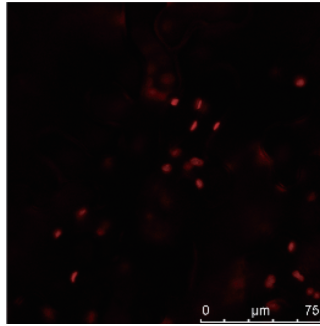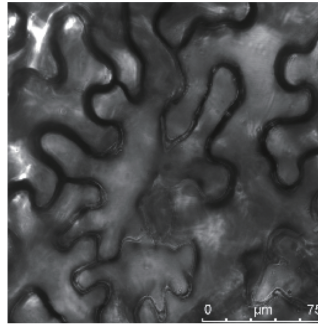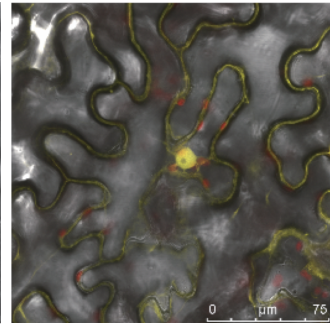

FreeYFP+  
Auto chl

Supplement: msae028_Supplementary_Data [file msae028_supplementary_data.zip › Supplementary Figure 6.pdf]

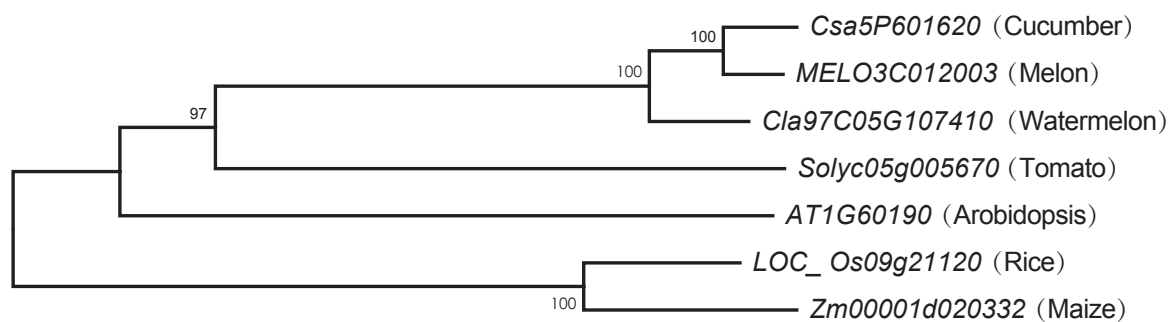

0.1

Supplement: msae028_Supplementary_Data [file msae028_supplementary_data.zip › Supplementary Figure 7.pdf]

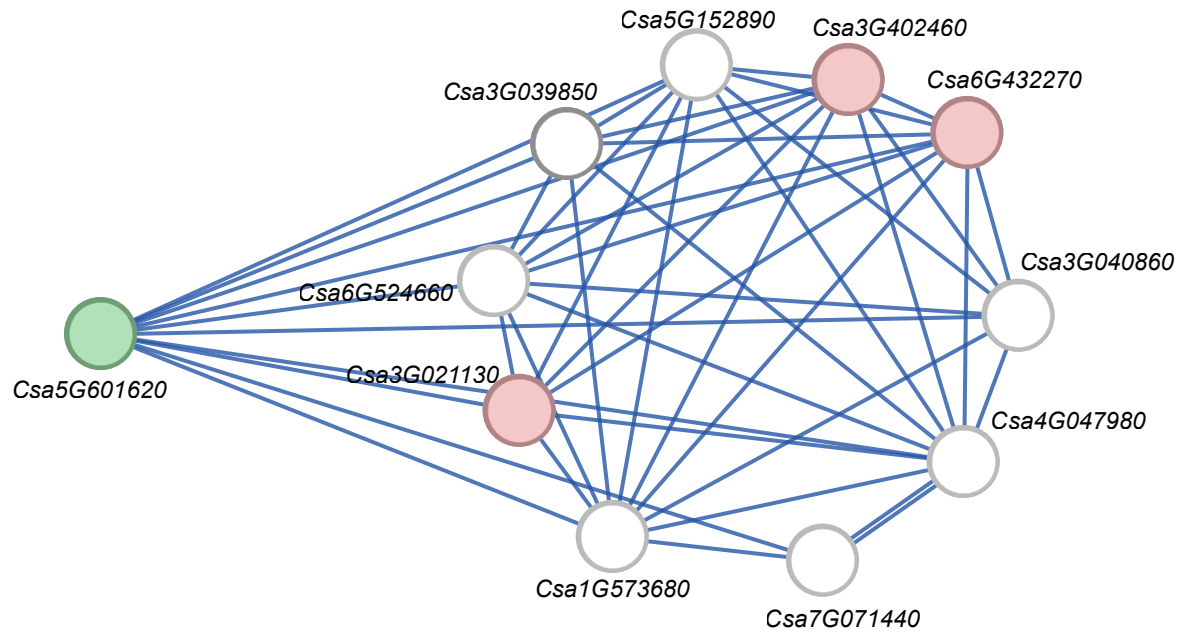

Supplement: msae028_Supplementary_Data [file msae028_supplementary_data.zip › Supplementary Figure 8.pdf]
